# Virtual Reality-Based Framework to Simulate Control Algorithms for Robotic Assistance and Rehabilitation Tasks through a Standing Wheelchair

**DOI:** 10.3390/s21155083

**Published:** 2021-07-27

**Authors:** Jessica S. Ortiz, Guillermo Palacios-Navarro, Víctor H. Andaluz, Bryan S. Guevara

**Affiliations:** 1 Department of Electronic Engineering and Communications, University of Zaragoza, 44003 Teruel, Spain; 2 Departamento de Eléctrica y Electrónica, Universidad de las Fuerzas Armadas ESPE, Sangolquí 171103, Ecuador; vhandaluz1@espe.edu.ec (V.H.A.); bsguevara@espe.edu.ec (B.S.G.)

**Keywords:** control algorithms, dynamic model, kinematic model, rehabilitation, robotic assistance, standing wheelchair, virtual reality

## Abstract

The implementation of control algorithms oriented to robotic assistance and rehabilitation tasks for people with motor disabilities has been of increasing interest in recent years. However, practical implementation cannot be carried out unless one has the real robotic system availability. To overcome this drawback, this article presents the development of an interactive virtual reality (VR)-based framework that allows one to simulate the execution of rehabilitation tasks and robotic assistance through a robotic standing wheelchair. The virtual environment developed considers the kinematic and dynamic model of the standing human–wheelchair system with a displaced center of mass, since it can be displaced for different reasons, e.g.,: bad posture, limb amputations, obesity, etc. The standing wheelchair autonomous control scheme has been implemented through the Full Simulation (FS) and Hardware in the Loop (HIL) techniques. Finally, the performance of the virtual control schemes has been shown by means of several experiments based on robotic assistance and rehabilitation for people with motor disabilities.

## 1. Introduction

There are thousands of people worldwide with some type of physical disability, some of them due to congenital or birth diseases and some others due to spinal injuries caused by accidents or age-related problems. For years, people with motor disabilities have been belittled by society, considered to be a burden [1,2]. Nowadays, we are more aware of the limitations that people with disabilities face when performing actions or tasks of everyday life, and different mechanical methods, techniques and devices that can facilitate this group of people to integrate into society have emerged [3]. Depending on the degree of motor disability that affects a person, the use of canes, walkers, chairs, among other manual mechanisms, allow people to move independently. However, there is a group of people with disabilities in lower and/or upper limbs, or with severe motor dysfunctions who cannot manipulate conventional mechanical devices [1,2,4]. This group of people require permanent assistance, i.e., they depend on a third person to manipulate the device, get out of bed, use the toilet; in short, to carry out any type of daily activity, thus generating dependence on their family, friends or caregivers [5].

Technology developed in the area of the rehabilitation of people with disabilities considers that technological development must create bio mechanisms capable of coexisting with others aimed at performing tasks in changing work environments, for which control in their manipulation and locomotion approaches has been analyzed by various researchers [4,6,7,8,9]. Currently, the fusion between mechanics, electronics and software has allowed the development of robotic devices that facilitate a person to perform safe movements, and as well as providing a certain degree of autonomy to the person by offering motor assistance. These systems are known as assistance robots [4,10,11]. Among the most common autonomous or semi-autonomous robotic mechanisms for the assistance and rehabilitation of people with motor disabilities, we can highlight the following: walkers, autonomous wheelchairs, standing wheelchairs and exoskeletons, among others [9,11,12].

We find in the literature several studies focused on developing control strategies that allow a person with a motor disability to maneuver a robotic wheelchair through: electromyography (EMG) signals that receive movement of the neck and arm muscles [13,14]; electrooculography (EOG) signals, where control depends on the user’s eye movement [15]; electroencephalography (EEG) signals which are used to define the movement of the robotic wheelchair [10,14]; or even control via voice command [16]. The abovementioned works are intended for allowing the user to move around in a partially structured environment. On the other hand, according to the activities of daily living (ADL) that a person may carry out, there is a need for the person with a motor disability to continuously change their sitting position, and vice versa [3]. In this context, great interest has been generated in the scientific community to develop prototypes of a robotic standing wheelchair, in order to improve the quality of life of people with motor disabilities [4,10]. Thus, different control algorithms are currently being proposed for the execution of autonomous or semi-autonomous tasks in partially structured and unstructured environments, through the standing human–wheelchair system. Autonomous care and rehabilitation are considered among the most common tasks found in the literature, so the implementation of control algorithms must ensure safe and reliable robotic systems for the user [4,17,18].

Therefore, the evaluation of the control algorithms requires a considerable number of experimental tests, in order to correct possible errors in accuracy and precision. However, this process cannot be easily carried out due to external factors, such as: (i) availability: people with disabilities have limited movement, which leads to a time conflict in participating in the required experimental tests; (ii) accident risk: people with motor disabilities who participate in experimental tests of any type of bio mechanism are exposed to possible accidents since the reactions to avoid blows, injuries and fractures are reduced compared to a person who does not have a motor disability; and finally, (iii) lack of bio mechanisms: the high costs of either bio mechanisms or elements for their construction limit researchers in carrying out experimental tests, as they are required to verify the correct operation of the designed controls algorithms [19].

As explained in previous paragraphs, the simulation of robotic applications for the assistance and rehabilitation of people with motor disabilities is an essential step prior to the experimental implementation of new research proposals. The main objective of the simulation is to recreate the real behavior of the patient when being subjected to assistance and rehabilitation tasks, without putting at risk the integrity of the person and the robotic system in the development stage. In addition, the implementation costs are radically reduced by dispensing with the physical robotic system until the end of the development process.

### Main Contributions of the Study

For all the above, and to overcome the different factors that prevent the implementation of control algorithms in a real robotic system, we think it is essential to implement different technological tools to solve this problem, in order to continue developing new research proposals aimed at autonomous or semi-autonomous control of the robotic system in the area of service robotics, specifically in the patient rehabilitation area. Therefore, in this work, the development of an interactive and virtual system was oriented to simulate advanced control strategies for rehabilitation tasks and robotic assistance for people with motor disabilities through a robotic standing wheelchair. Unlike the works available in the literature, the developed VR-based system considers the implementation of closed-loop control algorithms, through the techniques of FS and HIL. After reviewing the literature regarding the autonomous control of wheelchairs, it can be concluded that there are different works to solve the trajectory tracking problem, where the desired speed is equal to the derivative with respect to the time of the desired trajectory. In addition, we may find works where control strategies are implemented to solve the trajectory tracking problem, but when it comes to demonstrating the stability of the proposed controller, they consider the desired speed of the robotic system constant. The proposals found in the literature for the autonomous control of a standing chair are not the best. The movements of the chair–man system must not depend exclusively on the desired trajectory, nor must the speed of movement always be constant. Therefore, in this work, a control algorithm is proposed for the autonomous control of rehabilitation and robotic assistance tasks are based on solving the standing wheelchair path following problem defined in the axes with respect to the inertial reference system. The proposed controller considers that the desired speed of the standing wheelchair is variable and may depend on the parameters of the desired task or the vital signs of the person, which differs from the works found in the literature. On the other hand, the virtualized environment considers the kinematic and dynamic behavior of the standing human–wheelchair system; therefore, a dynamic model is proposed that considers the lateral displacement of the center of mass of the human–wheelchair system, which differs from works found in the literature. The lateral displacement of the center of mass can be generated by the bad posture of the person, amputation of limbs, or a person with spinal injury, among others. In addition, the kinematic model and the dynamic model consider as input signals the maneuverability velocities of the standing wheelchair, in a similar way to commercial robots. Another relevant difference is that in this work, the proposed virtual system considers the development of dynamic link libraries (DLLs) that generate shared memory (SM) in RAM. The SM allows the exchange of information, in real time, between the virtual system developed in the Unity 3D graphics engine and the MatLab mathematical software (the MathWorks Inc., Natick, MA, USA), in which the advanced control algorithm is implemented to simulate rehabilitation or robotic assistance tasks. Finally, the robustness of the proposed control scheme is mathematically analyzed, guaranteeing that the control errors are limited as a function of the velocity error. The velocity error is generated by the friction force between the robotic wheelchair and the selected surface in the virtual environment, thus resembling reality.

The article is organized as follows: Section 2 presents the state of the art, whereas Section 3 deals with the formulation of the problem and describes the proposal to be developed in this work. The kinematic and dynamic models featuring the robotic standing wheelchair velocities as inputs are presented in Section 4, whereas the development of the interactive and virtual environment is presented in Section 5. Section 6 deals with the design of the control algorithm for the execution of rehabilitation and autonomous assistance tasks, together with a robustness analysis of the proposed control scheme. The experimental results are presented in Section 7 and discussed in Section 8. Finally, Section 9 presents the main conclusions together with future work.

## 2. Review of Literature

Nowadays, the development of software that allows one to simulate work environments is booming, due to the interaction that it offers to the user with diverse multidisciplinary systems of certain complexity [20,21,22]. The purpose of work environments is to help and support the user during the fulfillment of a task, and also to evaluate the correct functioning of the system [22]. The technological advances of the last decade have allowed the expansion of the use of simulators in several areas, e.g., social sciences, engineering, robotics, medicine and rehabilitation, among others [23,24]. In the rehabilitation area, simulation software has become an ally because it allows the patient to perform a sequence of exercises in a more interactive way, avoiding the frustration and boredom that can be generated in the patient [25].

A review of the literature shows that there are simulators oriented to robotic applications and simulators for rehabilitation applications. (i) Commercial robotics simulators: among the commercial simulators applied to robotics are Gazebo, V-REP and Webots, among others [26]. The programming language of these simulators is mainly based on C++ and Phyton, and they are compatible with ROS (Robotic Operating System), which allows for direct communication with the scientific programming software Matlab. However, they lack the possibility of introducing the behavior of a human in the form of an avatar, a feature that is essential for research related to robotic assistance and rehabilitation; (ii) simulators for rehabilitation: among the main simulators under development oriented to rehabilitation tasks are: Development of Exergaming Simulator for Gym Training, a prototype simulator combines various gym and rehabilitation equipment (treadmill, exercise bike, etc.) with virtual environments, games, sports applications, immersive gaming view and advanced motion controllers [27]. For the cognitive rehabilitation process, the scientific community is developing different prototypes of robotic assistants that consider virtual reality, augmented reality and mixed reality [28,29,30]. In [29], a simulator considering a wheelchair for Parkinson’s tremor testing is presented. A rehabilitation process for the restoration of lower limb gait is presented in [30]. In the works found in the literature, the applications developed only consider the virtual environment as a 3D plotter, which does not consider the dynamics of movement of the human–robot system, nor does it allow the implementation of assistance or rehabilitation tasks autonomously.

Currently, Unity3D (Unity Software Inc., San Francisco, CA, USA) is one of the most widely used 3D graphics engines for the development of simulators for robotic applications and for physical–cognitive rehabilitation tasks [31]. The advantage of Unity 3D is the compatibility with different formats, low latency of data exchange in real time, versatility to interact with other software, integrated supports for video cards and support for VR devices [32,33]. In this context, virtual environments can be designed to enable people with motor disabilities to perform assistive and rehabilitative tasks considering activities of daily living. Virtual environments developed for rehabilitation and robotic assistance applications for people with motor disabilities should be interactive environments that allow implicit interaction and sensory immersion of the user, thus ensuring that the experience in the virtual environment is as similar as possible to the experience in the real world [34,35].

## 3. Problem Formulation

The control algorithms for any developed standing wheelchair robot must be evaluated through different experimental tests to verify their robustness, stability and efficiency. To accomplish this, it is essential to have the standing wheelchair robot. In many cases, this is a problem because the purchase or construction of the standing wheelchair represents a high cost for universities, research centers or companies focused on the development of assistance robots for people with physical disabilities. In addition, experimental tests are considered risky, since people with physical disabilities are exposed to some kind of accident. When evaluating the operation of the control algorithms, sudden movements can occur that may lead to blows, falls and injuries, because people with physical disabilities do not have the same reflexes and reaction skills to face these events as a person without physical disabilities. Table 1 presents the four alternatives for the implementation and evaluation of control schemes [32].

**Figure 1:**
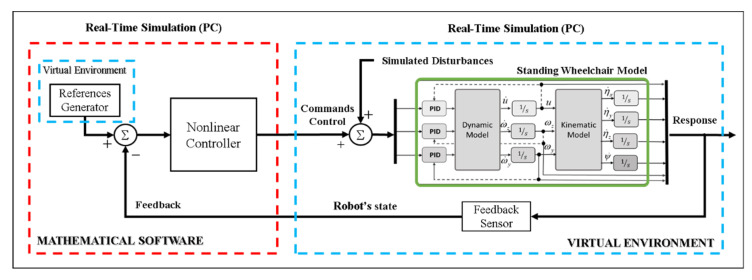
Block diagram for the FS technique.

**Figure 2:**
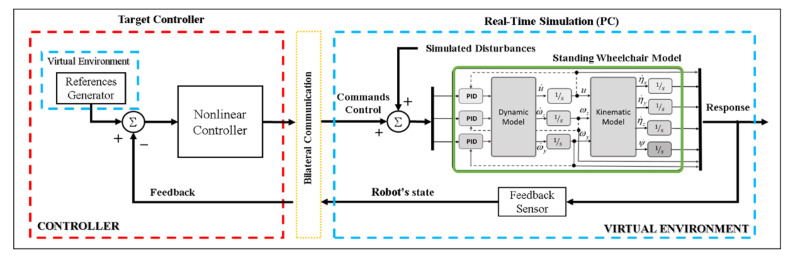
Block diagram for the HIL technique.

Due to the aforementioned drawbacks, when implementing closed-loop control algorithms with no possibility of having the robotic system, it is recommended to use a technique that emulates the real behavior of a robot–human system. Therefore, and considering that a robotic system is not available for the implementation and evaluation of control algorithms, this work proposes the implementation of control schemes based on the FS and HIL techniques, respectively, in order to implement and evaluate control schemes for the assistance and rehabilitation of people with motor disabilities via robotic wheelchairs. For the two proposed implementation techniques, the emulation of the human–wheelchair system in a 3D VR environment has been considered, as shown in Figure 1 and Figure 2, respectively.

Figure 1 shows the implemented control scheme considering the FS technique. The implementation considers two main parts that make up a closed-loop control scheme, defined as: (i) Target Controller: this block is within the mathematical software that allows the implementation of control algorithms, in charge of correcting control errors to accomplish the desired task to be performed; (ii) Virtual Environment: this block fulfills the function of simulating the behavior of a robotic system which interacts with a 3D virtual environment. This block considers the mathematical modeling that represents the kinematics and dynamics of the robotic system, including disturbances that affect the system (e.g., friction between the robot and the environment, noise at the input and output of the robotic system, among others).

Figure 2 details the three main parts that make up a closed-loop control scheme considering the HIL technique, defined as: (i) Target Controller: this contains the control algorithm in charge of correcting possible errors between the reference signal and the output; (ii) Real-time Simulation: this block fulfills the function of simulating the behavior of a robotic system, considering the mathematical modeling that represents both the kinematics and dynamics of the robotic system. In addition, this block can include disturbances that may affect the system and the sensor in charge of receiving the output signal; and (iii) Bilateral Communication: this is the communication channel in charge of communicating the real part of the process with the simulation part in real time.

FS and HIL techniques offer advantages in the process of implementing the control scheme, such as: reduced development times, evaluation of the robustness of the control algorithm against disturbances in the system, reliability in system data and analysis in the implementation of security protocols (essential for an assistance robot), among others. These techniques require the knowledge of both the kinematic and dynamic behavior of the robot–human system. Therefore, mathematical models are one of the main requirements to validate the correct operation of the control techniques to be implemented. It should be noted that the simulation of the robot–human system evolves in real time, through a system of differential equations, identified and validated with a real system.

## 4. Robotic Standing Wheelchair Modeling

**Figure 3:**
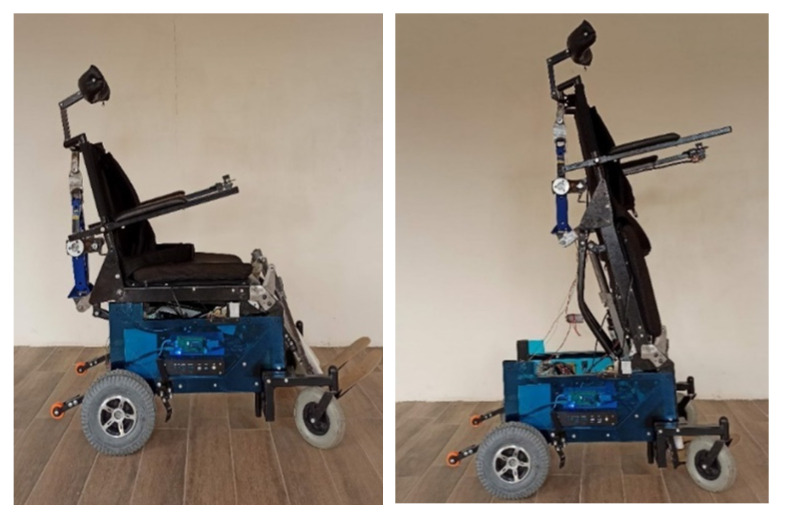
Robotic standing wheelchair built by the authors.

This section describes the modeling of the standing wheelchair (see Figure 3) in order to be implemented in the 3D simulator proposed in this work. This work considers the kinematic modeling of the wheelchair, as well as the dynamic model of the robotic system with displacement of the center of mass.

### 4.1. Kinematic Modeling

**Figure 4:**
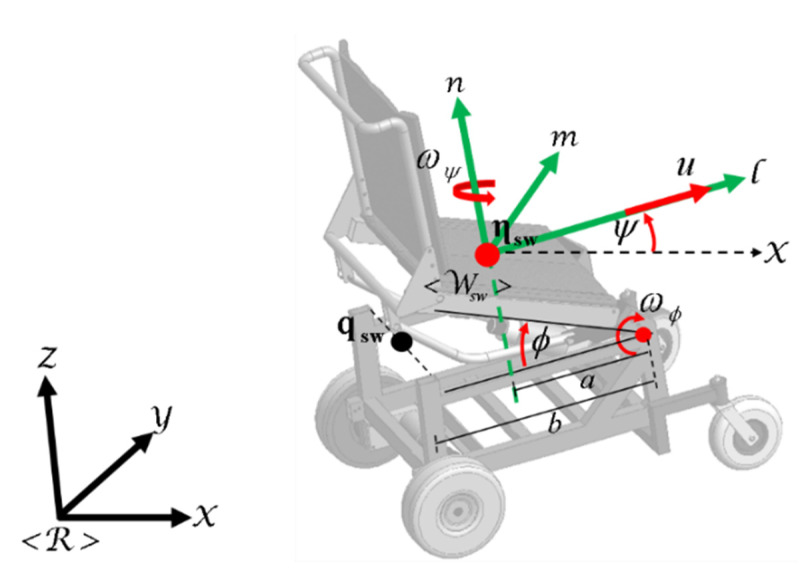
Robotic standing wheelchair.

This work is based on a non-holonomic mobile platform with standing. A robotic standing wheelchair is a differential drive mobile robot (DDMR) that can rotate freely around its vertical axis and move independently on the vertical axis. It is assumed that the human–wheelchair system with standing moves on (X,Y,Z) axis of a reference system <R>. The kinematic model of robot is confirmed by a set of three velocities represented at the spatial frame $<W_{sw}>$. The displacement of the robot is guided by a linear velocity u, and two angular velocities $\omega_{\psi }$ and $\omega_{\phi }$, as shown in Figure 4.

In other words, the Cartesian motion of the standing wheelchair robot at the inertial frame <R>, is defined as

(1) $$\left[\begin{array}{c} {\dot{\eta }}_{x}\\ {\dot{\eta }}_{y}\\ {\dot{\eta }}_{z}\\ \dot{\psi } \end{array}\right]=\left[\begin{array}{ccc} \cos\psi & -a\sin\psi -b\sin\psi +b\cos\phi \sin\psi & b\sin\phi \cos\psi \\ \sin\psi & a\cos\psi +b\cos\psi -b\cos\phi \cos\psi & b\sin\phi \sin\psi \\ 0 & 0 & b\cos\phi \\ 0 & 1 & 0 \end{array}\right]\left[\begin{array}{c} u\\ \omega_{\psi }\\ \omega_{\phi } \end{array}\right]\;{\dot{\eta }}_{sw}\left(t\right)=J\left(\psi ,\phi \right)\mu \left(t\right)$$

where a and b are distances; ${\dot{\eta }}_{x}$,${\dot{\eta }}_{y}$,${\dot{\eta }}_{z}$ and $\dot{\psi }$ are the point interest velocities (whose position is being controlled) with respect to the inertial frame <R>; $J\left(\psi ,\phi \right)\in R^{m\;x\;n}$ represents the Jacobian matrix that defines a linear mapping between the velocities vector ${\dot{\eta }}_{sw}\left(t\right)\in R^{m}$ with m=4 and of the standing wheelchair maneuverability velocities vector $\mu \left(t\right)\in R^{n}$ with n=3.

### 4.2. Standing Wheelchair Dynamic Model

**Figure 5:**
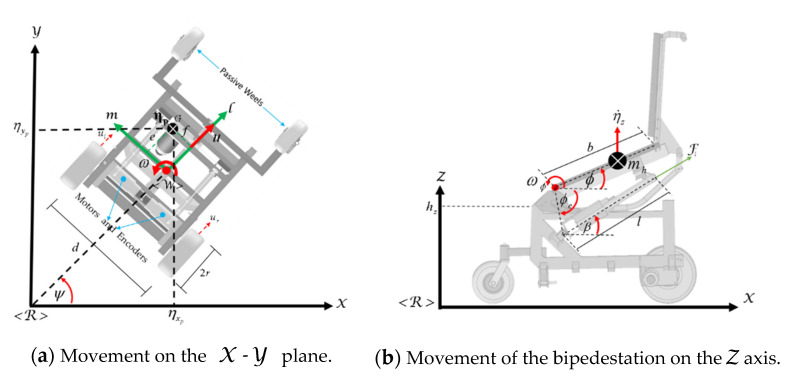
Schematic of the robotic standing wheelchair.

In this subsection, the dynamic modeling of the standing wheelchair robot is presented, for which a separate analysis is considered. For the dynamic model of the wheelchair without standing, it is assumed that the human–wheelchair system moves on a planar horizontal surface, where the vertical disturbances have been neglected, whereas for the dynamic model of standing, only linear motion about the Z axis is considered, as shown in Figure 5.

The dynamic model of a robotic system can be obtained through the force equilibrium approach established by Newton’s second law, or its equivalent for rotational movements, the so-called Euler’s law [4]. However, in this work, a simple and systematic conceptualization is considered through the kinetic and potential energy balance approach established by the Lagrange formulation [36].

The Lagrange formalism is used to derive the dynamic equations of the human–wheelchair system. In the case of the dynamic model of the wheelchair without standing the potential energy P(q)=0, because the trajectory of the wheelchair is constrained to the horizontal plane. Thus, the kinetic energy is given by,

(2) $$L=K=\frac{1}{2}\left(m_{w}+m_{h}\right)v^{2}+\frac{1}{2}I\omega_{\psi }^{2}$$

where $m=m_{w}+m_{h}$ represents the human–wheelchair system mass, in which $m_{h}$ is the human mass and $m_{w}$ is the wheelchair mass; $v^{2}={\dot{\eta }}_{x_{p}}^{2}+{\dot{\eta }}_{y_{p}}^{2}$ is the velocity of the wheelchair on the X−Y plane; I is the inertia moment of the wheelchair–human system.

On the other hand, for the dynamic model of standing, the Lagrangian equation is defined as,

(3) $$L=\frac{1}{2}m_{h}{\dot{\eta }}_{z}^{2}-m_{h}g\left(h_{z}+b\sin\left(\phi \right)\right)$$

where ${\dot{\eta }}_{z}\left(t\right)=\omega_{\phi }\left(t\right)b\cos\phi \left(t\right)$, $h_{z}$ is the constant height of the wheelchair seat.

Therefore, it is possible to obtain a dynamic model that considers both linear velocity and angular velocities as input signals, as commercial robots have [37].

(4) $$\left[\begin{array}{c} \mu_{ref_{p}}{\left(t\right)}_{2x1}\\ \omega_{\phi_{ref}}{\left(t\right)}_{1x1} \end{array}\right]=\left[\begin{array}{cc} M_{p}{\left(\varsigma \right)}_{2x2} & 0_{2x1}\\ 0_{1x2} & M_{b}{\left(\phi ,\varphi \right)}_{1x1} \end{array}\right]\left[\begin{array}{c} {\dot{\mu }}_{p}\\ {\dot{\omega }}_{\phi } \end{array}\right]+\left[\begin{array}{cc} C_{p}{\left(\varsigma ,\mu_{p}\right)}_{2x2} & 0_{2x1}\\ 0_{1x2} & C_{b}{\left(\phi ,\dot{\phi },\varphi ,\dot{\varphi }\right)}_{1x1} \end{array}\right]\left[\begin{array}{c} \mu_{p}\\ \omega_{\phi } \end{array}\right]+\left[\begin{array}{c} 0_{2x1}\\ g{\left(\phi \right)}_{1x1} \end{array}\right]\;\mu_{ref}\left(t\right)=M\left(\phi ,\varphi ,\varsigma \right)\dot{\mu }+C\left(\phi ,\dot{\phi },\varphi ,\varsigma ,\mu \right)\mu +g(\phi )$$

where $M\left(\phi ,\varphi ,\varsigma \right)\in R^{nxn}$ with n=3 represents the inertia matrix of the standing human–wheelchair system; $C\left(\varsigma ,\mu \right)\in R^{nxn}$ represents the centripetal and Coriolis forces; $g\left(\phi \right)\in R^{n}$ represents the gravitational vector; $\mu =[\begin{array}{ccc} u & \omega_{\psi } & \omega_{\phi } \end{array}]\in R^{n}$ is the vector of system’s velocity; and $\mu_{ref}=[\begin{array}{ccc} u_{ref} & \omega_{\psi_{ref}} & \omega_{\varphi_{ref}} \end{array}]\in R^{n}$ is the vector of velocity control signals for the standing human–wheelchair system; and $\varsigma =[\begin{array}{cc} \varsigma_{p} & \varsigma_{b} \end{array}]\in R^{l}$ with $l=l_{p}+l_{b}=22$ is the vector of dynamic parameters, which contain the physical, mechanical and electrical parameters of the human–wheelchair system. For more details on the dynamic model, see Ortiz’s proposal in [37]. Appendix A shows the dynamic parameters of the standing wheelchair.

## 5. Virtual Environment

Virtual environments intended for rehabilitation should consider a virtual environment that allows robot–human interaction with every day, real-life situations. Therefore, this section describes the development of a 3D virtual simulator that allows people with motor disabilities to perform autonomous rehabilitation and assistance tasks. The virtual environments developed are related to everyday tasks in a person’s real life, with the aim of evaluating the performance of closed-loop control algorithms in a more realistic way.

**Figure 6:**
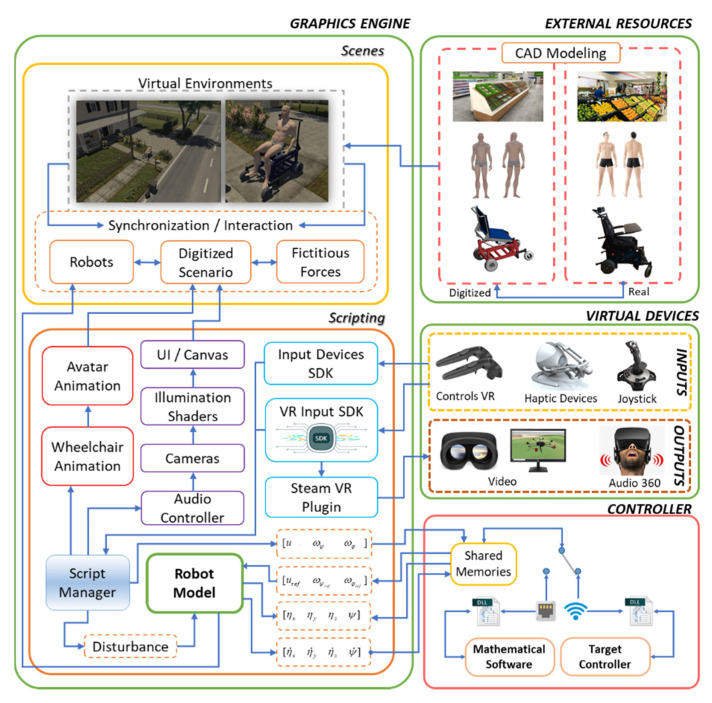
Proposed virtual environment schema.

The implementation scheme of the Virtual Standing Human–Wheelchair System simulator (VSWHS), is presented in Figure 6, which consists of external graphic resources that are executed on a Unity3D graphic engine. The proposed scheme consists of four main blocks: (i) external resources, which consider the development of 3D objects to be included in the virtual environment; (ii) 3D graphics engine, which contains the implementation of external resources and programming scripts that allow the simulation of robot–human interaction in a virtual environment; (iii) virtual devices, which allow user immersion and interaction with the virtual environment; and finally (iv) control algorithm, which allows the implementation of closed-loop control algorithms, in order to carry out rehabilitation or autonomous assistance tasks for people with motor disabilities.

### 5.1. External Resources

**Figure 7:**
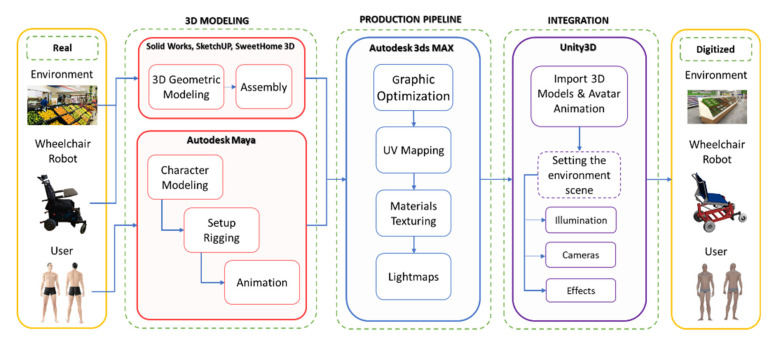
External resources virtualization.

External resources are essentially made up of three groups: (i) virtualized scenario, referring to scenarios related to ADL, to evaluate rehabilitation and autonomous assistance tasks for people with partial or total motor disabilities; (ii) virtualized robot, related to 3D modeling of the standing wheelchair and its assembly (it is carried out in the Solid Works (SolidWorks Corp., Waltham, MAassachusetts, USA) CAD software). This process is based on the dimensions and physical characteristics of a real wheelchair; and (iii) avatar, which represents the person or user who will be participating in the use of the simulator, and whose character is modeled in the Autodesk Maya software taking into account the anthropomorphic dimensions of the average individual (see Figure 7).

### 5.2. Graphics Engine

Unity has been considered as a 3D graphics engine. Unity is a multiplatform video game engine created by Unity Technologies. Unity is available as a development platform for Microsoft Windows, Mac OS, and Linux [32]. We separated the virtual environment development process into two main parts: 3D scene development and programming of the virtual environment control scripts, respectively.

#### 5.2.1. Virtual Scene

This subsection describes the 3D scenes developed for applications aimed to simulate rehabilitation proposals and robotic assistance. In addition, the proposed system considers the implementation of a user interface, which allows one to define the simulation parameters, e.g., desired task, virtual environment to execute the desired task and physical characteristics of the avatar, among others.

**Figure 8:**
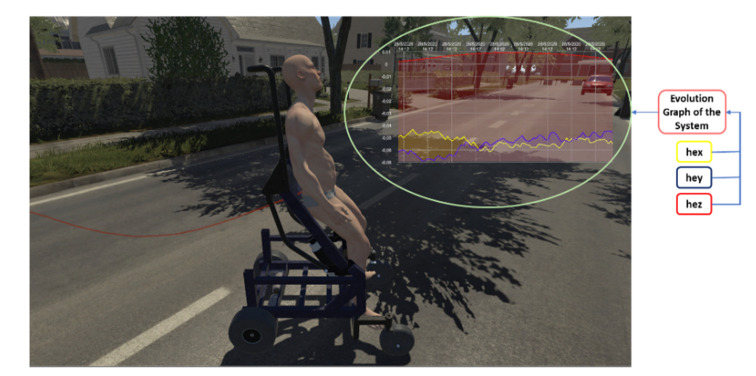
Data visualization of the control errors evolution in the graphics engine.

(a) User Interface (UI). This was developed to allow easy and intuitive interaction with the program to start up the virtual control scheme and allow the user to visualize the evolution of the system, as well as the data represented as variables of the states of the robot–human system. An important feature is that, depending on the dynamic disturbance data of the controller, the height and weight of the avatar can be modified to simulate in a more reliable and credible way the real behavior of the robot–human system. Another important detail is the development of a real-time graphics system that allows the visualization of the control errors evolution locally in the graphics engine without the need to pay attention to the scientific programming software (see Figure 8).

(b) Realism and Rendering. The development of 3D scenes is a fundamental process to create realistic virtual environments that are capable of deceiving the user’s senses. Thus, when importing external resources, it is necessary to make some virtualized environment and robot configurations.

The Meshing stage considers the data of the vertices and faces of the objects aimed at taking the geometry from the Mesh Filter and renders it at the position defined by the GameObject’s Transform component. The Material stage defines the textures, material properties, and the Lighting and Lightmapping components of the imported external resources. In order to optimize the graphic rendering performance in the Shaders stage, each external resource is customized through specialized scripts that contain mathematical algorithms that calculate the color of each rendered pixel based on the lighting input and the material configuration. Finally, these settings are stored in “Prefabs” for later use.

#### 5.2.2. Scripting Stage

**Figure 9:**
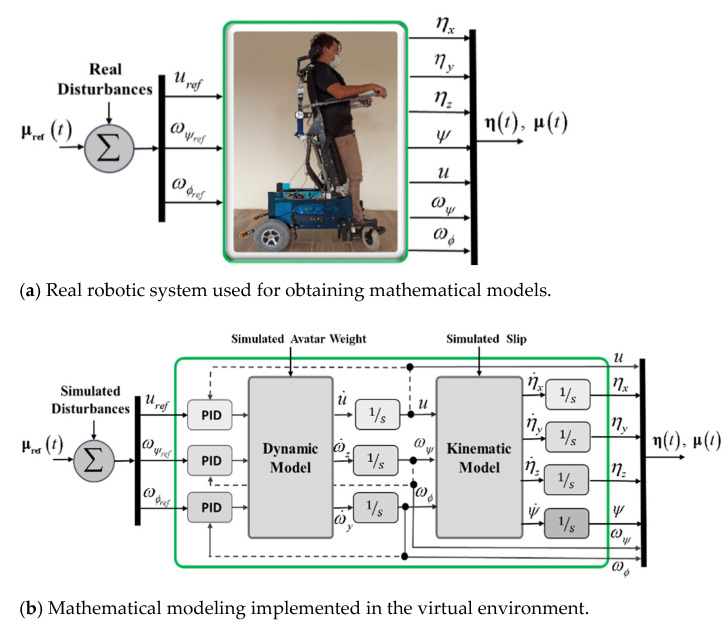
Cinematic and dynamic behavior of the human–robot system.

One of the functionalities defined as a set of the most relevant public classes when implementing a 3D virtual environment that allows one to emulate the behavior of a robot–human system, is the kinematic and dynamic modeling block of the robotic system. It should be noted that the proposed dynamic model (Equation (4)) allows one to modify the avatar weight and considering external disturbances, which can be generated by sliding on smooth surfaces, or by the noise generated at the inputs of the maneuverability commands and at the outputs of the robot, for example. The sliding of the wheels is affected by the friction forces that are generated according to the type of soil in the virtual environment where the wheelchair performs the desired task. Figure 9 shows the model block of the robotic system considered in this work, where both the mathematical models representing a wheelchair and the actual robotic system consider the same input and output signals.

**Figure 10:**
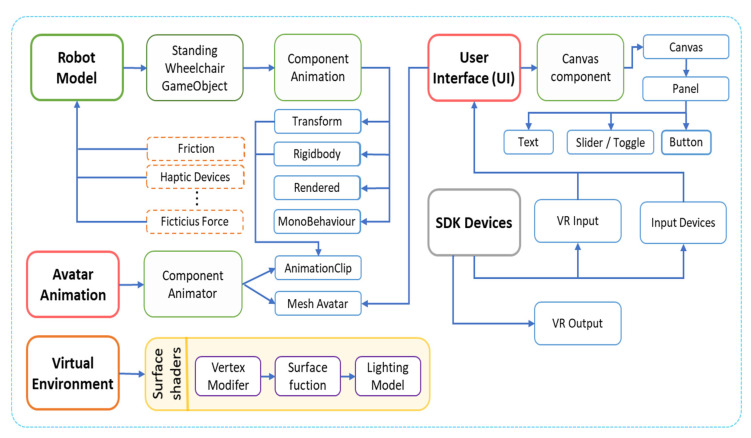
Scripting general scheme.

On the other hand, the scripts contain the code blocks with the necessary instructions that determine the functionality of a set of tools, data and components that make up the 3D virtual simulator. In this layer, the dedicated libraries (SDK—Software Development Kit) of the virtual input and output devices are managed, which allow communication and interaction with each other. In addition, these blocks manage the components involved in the scene, such as the robotic system model, the audio controller, cameras, lighting, user interface (UI) and the generation of fictitious forces that, together, simulate real conditions which robots are subjected to during operation (see Figure 10).

Through the dynamic modification of the mesh of the avatar model in its masculine and feminine version, it is possible to modify the physical appearance representing the accumulation of fat based on the configured weight. In the same way, it is also possible to modify the height, maintaining an anthropomorphic proportion of the human body. At this stage, animation of the movement of the robotic wheelchair is also performed based on the workspace that is defined by the control algorithm. In a similar way, the animation frames of the avatar are synchronized according to the state variables of the standing wheelchair.

### 5.3. Inter-Process Communication—Shared Memory

**Figure 11:**
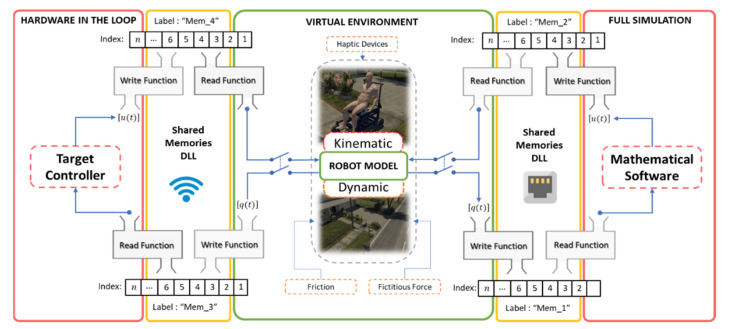
Data exchange between virtual environment and destination controller.

The exchange of information between memory segments is a feature of operating systems, that enables one to share information. Taking into account the information provided by [27], in this work, we implemented the shared memory method, since it is an easy technique to apply, with short delays and low computational cost because no third party functions are used. Figure 11 presents the data exchange scheme based on shared memory, proposed in this work. For the FS technique, data exchange is considered between the 3D simulator that is developed in the Unity graphics engine, and the mathematical software in which the wheelchair control algorithm is implemented. On the other hand, for the HIL technique, data exchange is considered between Unity and the target hardware in which the wheelchair control algorithm is implemented.

**Figure 12:**
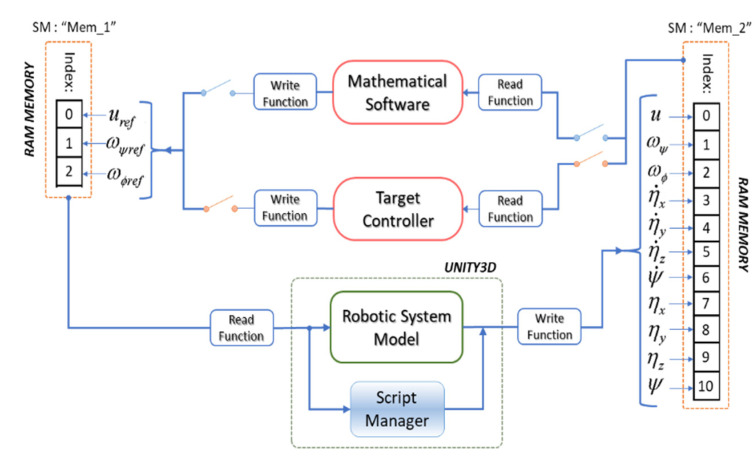
Publisher/subscriber of the shared memories.

Figure 12 contains scripts that allow the exchange of information between the virtual environments with mathematical software, through the use of a dynamic link library (DLL) that generates a shared memory in RAM (SM) for the exchange of data between different software packages. By means of the SM, the control actions calculated in the destination controller are injected into the mathematical model of the robotic system. The model of the robotic system calculates its position and velocity outputs, which are sent to the mathematical software, thus closing the control loop through the feedback of the robot’s output states.

## 6. Control Algorithm Design

The proposed control algorithm for the execution of rehabilitation and autonomous assistance tasks must be implemented according to the technique to be used. That is, for the FS technique, a different mathematical software hosted on the same computer as the virtual environment is considered. Regarding the HIL technique, a hardware of a different kind than the computer where the 3D virtual environment is hosted is considered. On the other hand, with the aim of executing autonomous rehabilitation or robotic assistance tasks for people with motor disabilities, an advanced control algorithm is proposed to solve the problem of following the desired path $P\left(s\right)\in R^{3}$, not parameterized in time, defined on (X,Y,Z) axis of a inertial reference frame <R>.

**Figure 13:**
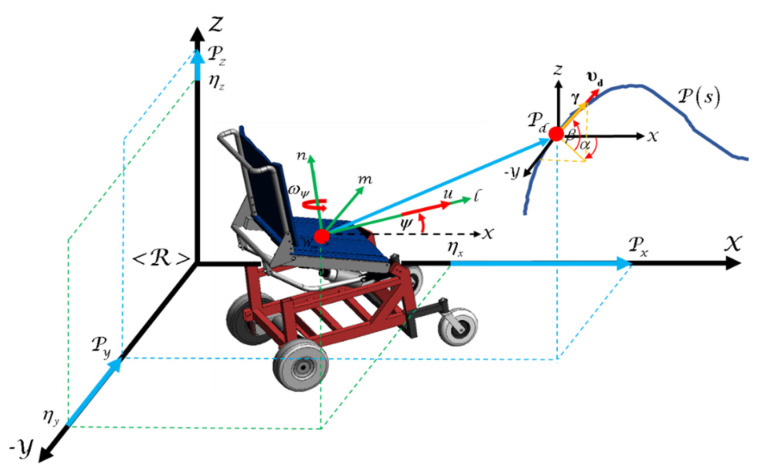
Path-following problem for a robotic standing wheelchair.

**Figure 14:**
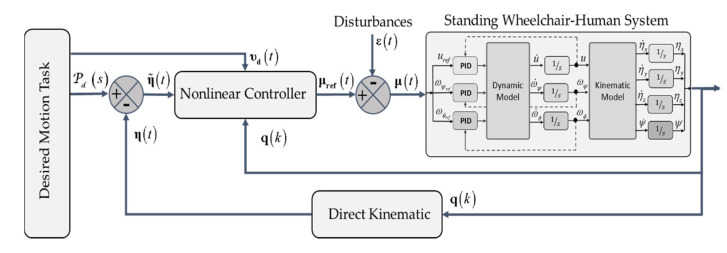
Block diagram of the motion control of the standing wheelchair-human system.

Figure 13 shows the wheelchair path-following problem, where $P_{d}=[\begin{array}{ccc} P_{x} & P_{y} & P_{z} \end{array}]$$\in R^{3}$ defines the closest point between the standing wheelchair and the desired path P(s). In addition, it is considered that the desired velocity of the wheelchair can be variable, which differs from works found in the literature, in which it is considered that the desired velocity is constant. In this work, the velocity can be defined according to the characteristics of the desired task, i.e., $\upsilon_{d}\left(t\right)=f\left(\upsilon_{\max},P,\eta \right)$. The proposed control algorithm will consider a non-linear control law based on the kinematic model of the robotic standing wheelchair (see Figure 14).

The proposed controller considers the saturation of the $\mu_{\min}<\mu_{ref}\left(t\right)<\mu_{\max}$ velocity commands, and receives as input signals $P\left(s\right)|s\in [s_{0},s_{f}]$, which describe the desired motion task of the standing wheelchair, respective to the inertial frame R(X,Y,Z). The problem of control to deal with—often called the inverse kinematics problem—is finding the control vector of maneuverability $\mu_{ref}\left(t\right)|t\in [t_{0},t_{f}].$ to achieve the desired operational motion. The corresponding evolution of the whole system is given by the actual generalized motion $q\left(t\right)|t\in [t_{0},t_{f}].$ Hence, the control error is defined as $\tilde{\eta }\left(t\right)=P_{d}\left(s\right)-\eta \left(t\right)$, and consequently, the control aim is expressed as $\underset{t\to \infty }{\lim}\tilde{\eta }\left(t\right)=0$$\in R^{m}$. The desired velocity of the standing wheelchair will depend on the task, the control error, the angular velocity, etc. In this case, it is considered that the reference velocity depends on the control errors and the angular velocity. It is defined as [4]:

(5) $$\left|\upsilon_{d}\right|=\frac{v_{\max}}{1+k_{\tilde{\eta }}\left\|\tilde{\eta }\right\|+k_{\Gamma }\left\|\Gamma_{P}\right\|}$$

where $v_{\max}$ is the desired maximum velocity on the desired path P(s); $k_{\tilde{\eta }}$ and $k_{\Gamma }$ are positive constants that are control error and radius of curvature of P(s), respectively. The radius of curvature is defined as [38],

(6) $$\Gamma_{P}\left(t\right)=\frac{\left\|\dot{P}\times \ddot{P}\right\|}{{\left\|\dot{P}\right\|}^{3}}.$$

The proposed control scheme considers the kinematics of the standing wheelchair represented by Equation (1), without considering the variation of the orientation, since due to its mechanical configuration, the wheelchair is oriented tangentially to the desired path profile:

(7) $$\left[\begin{array}{c} {\dot{\eta }}_{x}\\ {\dot{\eta }}_{y}\\ {\dot{\eta }}_{z} \end{array}\right]=\left[\begin{array}{ccc} \cos\psi & -a\sin\psi -b\sin\psi +b\cos\phi \sin\psi & b\sin\phi \cos\psi \\ \sin\psi & a\cos\psi +b\cos\psi -b\cos\phi \cos\psi & b\sin\phi \sin\psi \\ 0 & 0 & b\cos\phi \end{array}\right]\left[\begin{array}{c} u\\ \omega_{\psi }\\ \omega_{\phi } \end{array}\right]\;\dot{\eta }\left(t\right)=J_{sw}\left(\psi ,\phi \right)\mu \left(t\right)$$

Thus, the following control law is proposed for the standing wheelchair robot:

(8) $$\mu_{ref}\left(t\right)=J_{sw}^{-1}\left(\upsilon_{d}\left(t\right)+\Gamma \tanh\left(\Gamma^{-1}\kappa \tilde{\eta }\left(t\right)\right)\right)$$

where $J_{sw}^{-1}$ is the inverse Jacobian matrix of $J_{sw}\left(\psi ,\phi \right)$; κ and Γ are the definite positive diagonal matrices that weigh the control error $\tilde{\eta }\left(t\right)=P_{d}\left(s\right)-\eta \left(t\right)$. In order to include an analytical saturation of velocities in the standing wheelchair robot, the tanh(.) function, which limits the control errors $\tilde{\eta }\left(t\right)$ is proposed. The expressions tanh(.) denote a component by component operation. Additionally, $\upsilon_{d}\left(t\right)$ represents the desired velocities vector on the desired path:

(9) $$\upsilon_{d}\left(t\right)=\left[\begin{array}{c} v_{x}\\ v_{y}\\ v_{z} \end{array}\right]=\left[\begin{array}{c} \left|\upsilon_{d}\right|\cos\left(\beta \right)\cos\left(\alpha \right)\\ \left|\upsilon_{d}\right|\cos\left(\beta \right)\sin\left(\alpha \right)\\ \left|\upsilon_{d}\right|\sin\left(\beta \right) \end{array}\right].$$

The vector $\left|\upsilon_{d}\right|$ represents the modulus of the desired velocity; $v_{x}$, $v_{y}$ and $v_{z}$ are the projections of $\upsilon_{d}$ on the direction of the X, Y and Z axes, respectively, while α represents the orientation of the projection of γ on the X−Y plane measured from the X axis of the <R> reference system; and β is the angle between the tangent vector γ with the X−Y plane The angles are determined by:

(10) $$\alpha \left(t\right)=\tan^{-1}\left(\frac{{\dot{P}}_{y}}{{\dot{P}}_{x}}\right)\;\mathrm{and}\;\beta \left(t\right)=\tan^{-1}\left(\frac{{\dot{P}}_{z}}{\left\|\left({\dot{P}}_{x},{\dot{P}}_{y}\right)\right\|}\right).$$

### Robustness Analysis

The behavior of the control error of the interest point of the standing wheelchair is analyzed considering errors in velocity tracking, i.e., $\varepsilon \left(t\right)=\tilde{\mu }\left(t\right)=\mu_{ref}\left(t\right)-\mu \left(t\right)$. The velocity error can be caused by unwanted disturbances on the robotic chair. Therefore, by substituting Equation (8) in (7), the close loop equation is obtained:

(11) $$\upsilon_{d}\left(t\right)=\dot{\eta }\left(t\right)-\Gamma \tanh\left(\Gamma^{-1}\kappa \tilde{\eta }\left(t\right)\right)+J_{sw}\tilde{\mu }\left(t\right).$$

Remember that the desired velocity vector $\upsilon_{d}\left(t\right)$ is different from the time derivative of the desired path. Now, defining difference signal γ(t) as $\gamma \left(t\right)=\frac{d}{dt}P\left(s\right)-\upsilon_{d}\left(t\right)$ and remembering that $\dot{\tilde{\eta }}\left(t\right)=\frac{d}{dt}P\left(s\right)-\dot{\eta }\left(t\right)$ Equation (11) can be written as:

(12) $$\dot{\tilde{\eta }}\left(t\right)=\gamma \left(t\right)-\Gamma \tanh\left(\Gamma^{-1}\kappa \tilde{\eta }\left(t\right)\right)+J_{sw}\tilde{\mu }\left(t\right)$$

**Remark** **1.** *The desired velocity vector*$\upsilon_{d}\left(t\right)$*is tangent to the desired path*P(s) *and is collinear to the vector of the derivative of the desired path. Then,*γ(t)*is also a collinear vector to*$\upsilon_{d}\left(t\right)$*and*$\frac{d}{dt}P\left(s\right)$.

For the robustness analysis, the following Lyapunov candidate function is considered: $V\left(\tilde{\eta }\left(t\right)\right)=\frac{1}{2}{\tilde{\eta }}^{T}\tilde{\eta }$. Its time derivative on the trajectories of the system is, $\dot{V}\left(\tilde{\eta }\left(t\right)\right)={\tilde{\eta }}^{T}\gamma -{\tilde{\eta }}^{T}\Gamma \tanh\left(\Gamma^{-1}\kappa \tilde{\eta }\right)+{\tilde{\eta }}^{T}J\tilde{\mu }\left(t\right)$. A sufficient condition for $\dot{V}\left(\tilde{\eta }\left(t\right)\right)$ to be negative definite is,

(13) $$\left|{\tilde{\eta }}^{T}\Gamma \tanh\left(\Gamma^{-1}\kappa \tilde{\eta }\right)\right|>\left|{\tilde{\eta }}^{T}\left(\gamma +J_{sw}\tilde{\mu }\left(t\right)\right)\right|$$

For large values of $\tilde{\eta }\left(t\right)$, the condition in the Equation (13) can be reinforced as, $\left\|{\tilde{\eta }}^{T}\right\|\left\|\Gamma \tanh\left(\Gamma^{-1}\kappa \tilde{\eta }\right)\right\|>\left\|{\tilde{\eta }}^{T}\right\|\left\|\gamma +J_{sw}\tilde{\mu }\left(t\right)\right\|$. Then, $\dot{V}\left(\tilde{\eta }\left(t\right)\right)$ will be negative definite only if $\Gamma >\left\|\gamma +J_{sw}\tilde{\mu }\left(t\right)\right\|/\tanh\left(\Gamma^{-1}\kappa \tilde{\eta }\right)$. Hence, the control errors $\tilde{\eta }\left(t\right)$ decrease, while for small errors values of $\tilde{\eta }\left(t\right)$, the error is ultimately bound by:

(14) $$\left\|\tilde{\eta }\right\|<\frac{\kappa_{aux}\left\|J_{sw}\tilde{\mu }\left(t\right)+\gamma \left(t\right)\right\|}{\varsigma \lambda_{\min}\left(\kappa \right)\tanh\left(\kappa_{aux}\right)};\mathrm{with}\;0<\varsigma <1$$

If the velocity errors are bound, then, it can be concluded that the control error is also ultimately bound by Equation (14). The velocity error is generated by the frictional forces between the wheelchair and the surface where the desired task is being performed. The friction forces change according to the coefficient of friction between surfaces; therefore, the velocity error is different from zero, but it is bound $\left\|\tilde{\mu }\left(t\right)\right\|<k_{\tilde{\mu }}$, with $k_{\tilde{\mu }}$ being a positive constant.

## 7. Experimental Results

This section presents the results obtained from the developed virtual environment and the proposed control scheme. This section is divided into four parts. First, we introduce the virtual simulator with the interactive windows that allow the configuration of the VR environment and the physical characteristics of the avatar. Second, we present the results obtained from the implementation of the advanced control algorithms for autonomous rehabilitation and robotic assistance tasks (HIL and FS simulation techniques are considered in the tests). Third, we present the hardware performance and computational cost of the computer when running the developed virtual environment. Finally, the results of a usability test are presented, for a group of 20 people who experimented with the developed virtual system.

### 7.1. Virtual Human–Wheelchair System Simulator

This subsection presents the user interface (UI) developed in this work. In addition, the configuration of the virtual simulator for the execution of rehabilitation tasks and robotic assistance for people with motor disabilities is shown. The UI allows one to navigate through a series of windows that allow one to modify and store information about the executed task.

**Figure 15:**
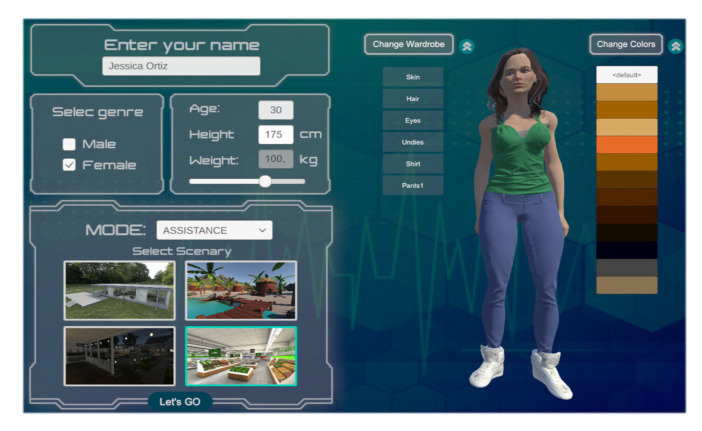
Scene configuration of the simulator environment for robotic assistance (initial scene).

Figure 15 shows the configuration scene of the informative data of the avatars that are used in the execution of the virtual desired tasks, e.g., name, gender, age, height and weight. The configuration of all the data enables the customization of the appearance of the avatar, with options including skin, hair, eyes, underwear, shirt and pants; each one with the possibility of modifying the type of material that determines the texture and color of the object. In addition, the configuration scene allows one to select the virtualized scenario where the experiment will be carried out. For this project, four available scenarios were developed.

**Figure 16:**
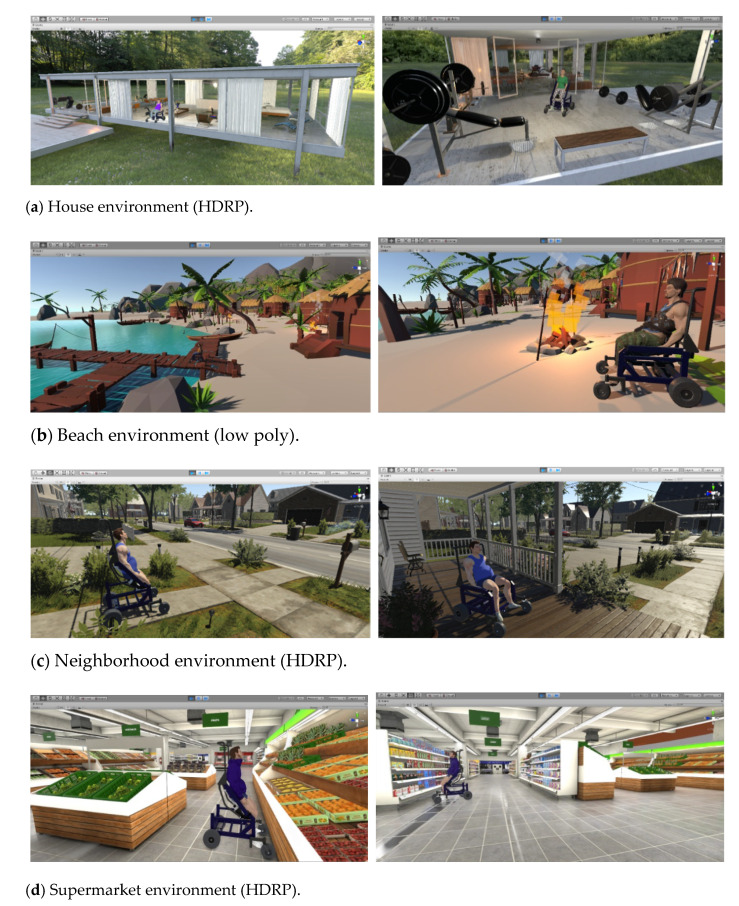
Screenshots of the developed virtual environments for the execution of rehabilitation tasks and robotic assistance, all related to activities of daily living.

Different virtual scenarios showing for the activities of daily living were developed to carry out autonomous assistance tasks. Figure 16 shows the virtualized scenarios, for which two types of visual art were considered: (i) High Definition Render Pipeline (HDRP), in which advanced visual optimization and lighting techniques were implemented to emulate the visual stimuli as faithfully as possible, as perceived in the real world; and (ii) Low poly style, which uses a small number of polygons in 3D models, with the purpose of seeking the abstraction of the elements and that the form takes over the design in such a way that a minimalist appearance is generated that encourages the user’s creativity to a certain extent during the execution of the experiment.

### 7.2. Control Scheme Implementation

This subsection shows the behavior of the implemented control schemes (based on the FS and HIL techniques described in Section 3 through experimental tests. In the implementation, the real-time interaction between human–wheelchair and the virtual environment was considered. The mathematical modeling of the human–wheelchair system presented in Section 4 and the development of the virtual environment presented in Section 5 were taken into account for virtual interaction. The control algorithm proposed in Section 6 was implemented in the target Hardware, according to the aforementioned techniques (HIL or FS).

#### 7.2.1. Experiment 1

**Figure 17:**
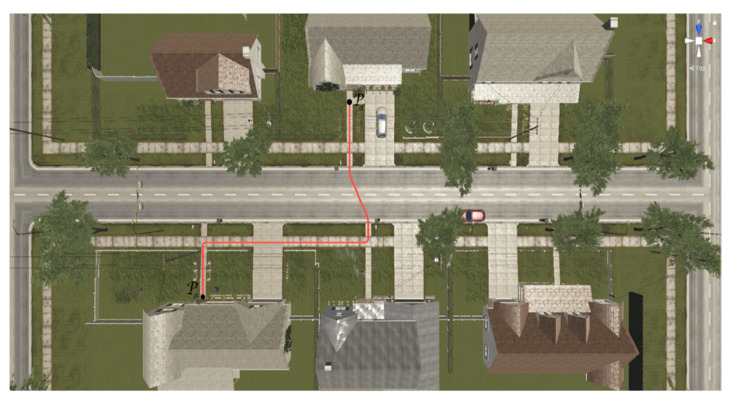
Autonomous assistance task: movement of the standing human–wheelchair system from a house located in $P_{o}$ to another house located in point $P_{d}$.

The first experiment considers the implementation of the FS technique, aimed at executing an autonomous assistance task. A male avatar was configured with an age of 35 years, a height of 1.75 (m), and a weight of 100 (kg). This information was included in the dynamic model of the standing wheelchair–human system represented by Equation (4). Additionally, the HDRP virtual environment representing a neighborhood environment was considered (see Figure 16c). For this experiment, the aim was to follow a desired path that allowed the autonomous displacement of the human–wheelchair system from an initial position $P_{o}$ to a final position $P_{d}$. The desired task was selected since the transfer of a person between two points is a common action of daily life. Figure 17 shows the desired path for the human–wheelchair system, obtained from the virtual scenario through a non-linear regression that determines the values of the parameters associated with the best fit curve.

Once the desired path was obtained, the desired path vectors were defined $\eta_{dx}$ and $\eta_{dy}$ with respect to the X−Y plane of the inertial reference system R(X,Y,Z). A constant posture was considered for the movement of standing on the Z axis, defined by $\eta_{dz}=0.5\;[m]$, the value representing the distance from the point of interest of control to the ground, i.e., a value that corresponds to the status of the avatar sitting in the wheelchair while executing the desired task. For the autonomous task execution, the control law proposed in Equation (8) was implemented, where the controller parameters were defined as: initial conditions of the robot $\eta_{o}=[\begin{array}{ccc} -54 & -3 & 0.85 \end{array}][m];$ desired path $\eta_{d}=[\begin{array}{ccc} \eta_{dx} & \eta_{dy} & \eta_{dz} \end{array}]$; weight matrix of control errors Γ=diag(1.8, 1.8, 1) and κ=diag(1.1, 1.1, 0.5). A sampling time of $T_{0}=0.1\;[s]$ was set.

**Figure 18:**
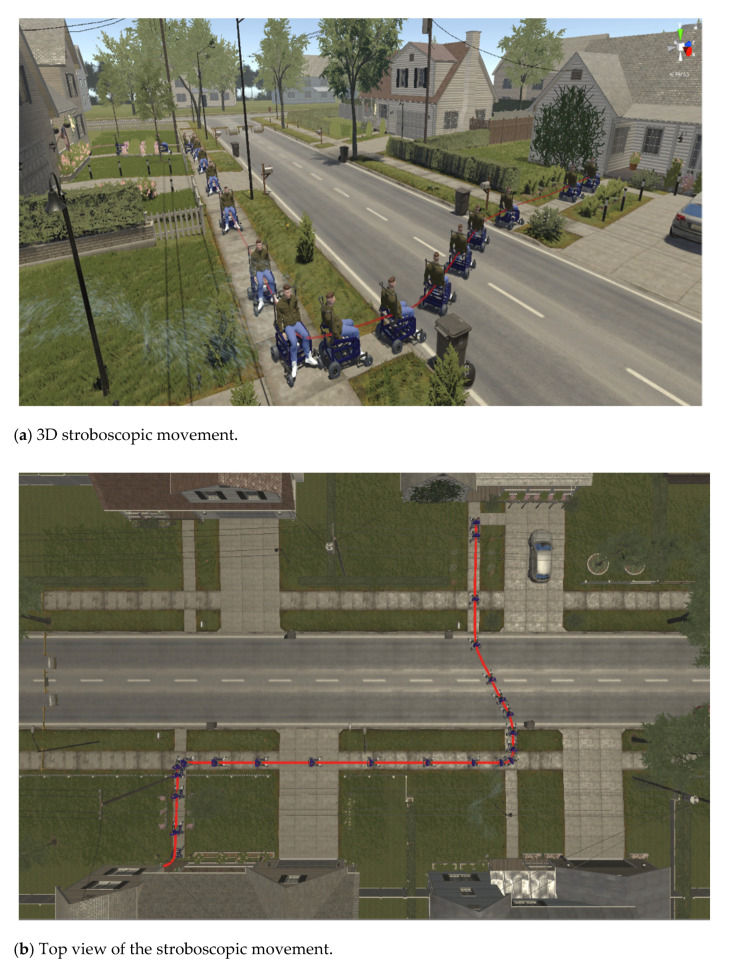
Virtual stroboscopic movement of the robot–human system based on the experimental data.

**Figure 19:**
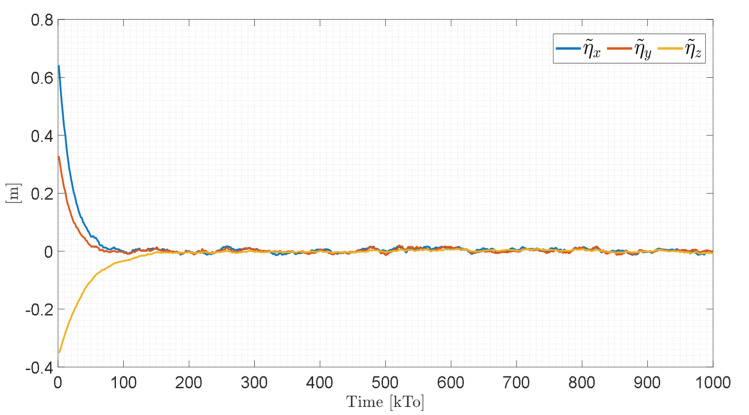
Time evolution of the control errors $\tilde{\eta }\left(t\right)=\left({\tilde{\eta }}_{x},{\tilde{\eta }}_{y},{\tilde{\eta }}_{z}\right)$**.**

**Figure 20:**
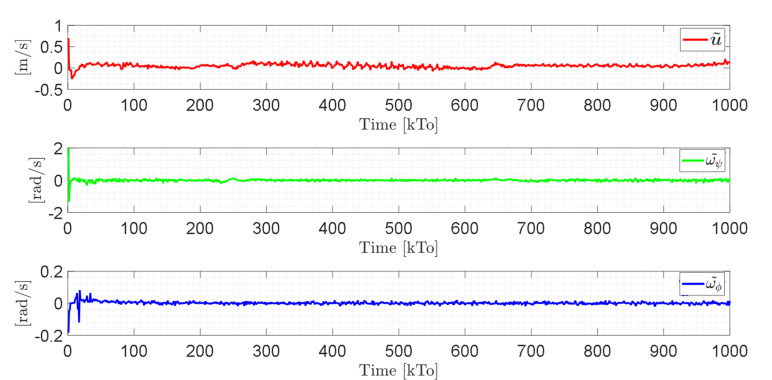
Time evolution of the velocity errors $\tilde{\mu }\left(kT_{0}\right)=\left(\tilde{u},{\tilde{\omega }}_{\psi },{\tilde{\omega }}_{\phi }\right)$.

**Figure 21:**
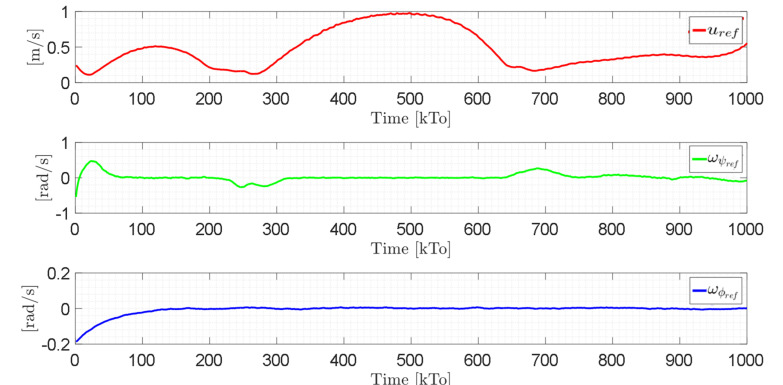
Velocity commands to the standing wheelchair $\mu_{ref}\left(kT_{0}\right)=\left(u_{ref},\omega_{\psi_{ref}},\omega_{\phi_{ref}}\right)$.

The evaluation of the autonomous assistance task was carried out through the analysis of the response curves of the proposed control algorithm. Figure 18, Figure 19, Figure 20 and Figure 21 show the results of the first experiment. Figure 18 shows the virtual stroboscopic movement of the robot–human system, based on real data.

Figure 19 shows that the control errors $\tilde{\eta }\left({\tilde{\eta }}_{x},{\tilde{\eta }}_{y},{\tilde{\eta }}_{z}\right)\in R^{3}$ converge to values close to zero asymptotically, i.e., achieving final feature errors $\max\left|\tilde{\eta }\left(t\right)\right|<0.04\;[m]$, since the velocity errors are bounded and different from zero $\tilde{\mu }\left(t\right)=\mu_{ref}\left(t\right)-\mu \left(t\right)\neq 0\in R^{3}$, as shown in Figure 20.

Figure 21 shows the control actions injected into the standing wheelchair robot during the experimental test. From the results obtained, the adequate performance of the proposed controller was verified.

#### 7.2.2. Experiment 2

The second experiment considers the implementation of the HIL technique. A female avatar was configured with an age of 21 years, a height of 1.6 (m) and a weight of 67 (kg). This information was included in the dynamic model of the human–wheelchair system represented by Equation (4). In addition, we used the virtual environment that represents a house (Figure 19). The experiment considered a task applied to autonomous rehabilitation routines, in which the standing movement is performed sinusoidally. The desired movement was considered a low frequency of movement, in order not to cause abrupt movements to the patient or unwanted injuries. For people with motor disabilities in their lower extremities, standing physical exercises are performed with the purpose of not losing muscle mass, reducing spasticity, preventing the appearance of ulcers, and it is even fundamental for physiological and social reasons, and to guarantee the correct development of the hip joint during childhood. Therefore, standing upright is key to avoid motor impairment in the case of neurological injuries or physical disability [39]. The desired task, desired velocity and initial conditions for the controller are defined in Table 2 for the experiment.

**Figure 22:**
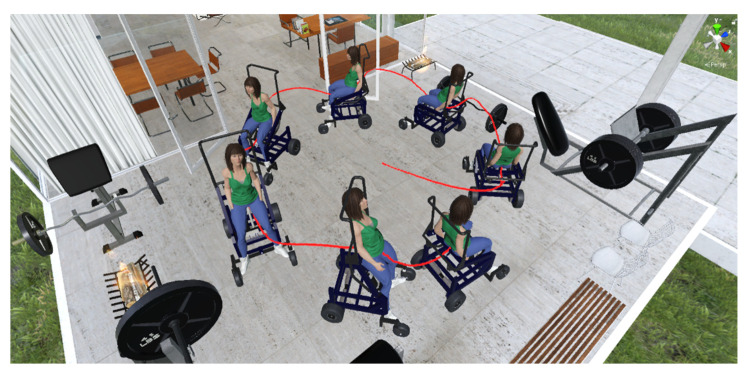
Virtual stroboscopic movement of the robot–human system.

Unlike the first experiment, the movement of standing in the Z axis was variable, while the displacement was executed with respect to the X−Y plane of the inertial reference system R(X,Y,Z). For the autonomous task execution, the same control law proposed in Equation (8) was implemented, where the controller parameters are defined as: the weight matrices of control errors Γ=diag(1.8, 1.8, 1) and κ=diag(1.1, 1.1, 0.5); the gain constants to define the desired velocity based on the desired task $k_{\tilde{\eta }}=1.4$ and $k_{\Gamma }=1.3$. Finally, a sampling time of $T_{0}=0.1\;[s]$ was set. Figure 22 shows the virtual stroboscopic movement of the robot–human system, based on real data.

**Figure 23:**
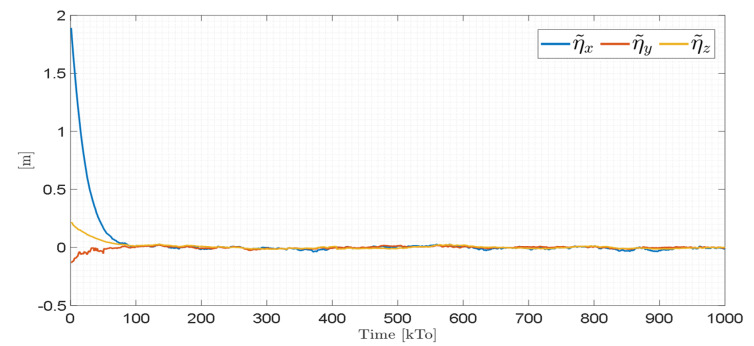
Time evolution of the control errors $\tilde{\eta }\left(t\right)=\left({\tilde{\eta }}_{x},{\tilde{\eta }}_{y},{\tilde{\eta }}_{z}\right)$.

**Figure 24:**
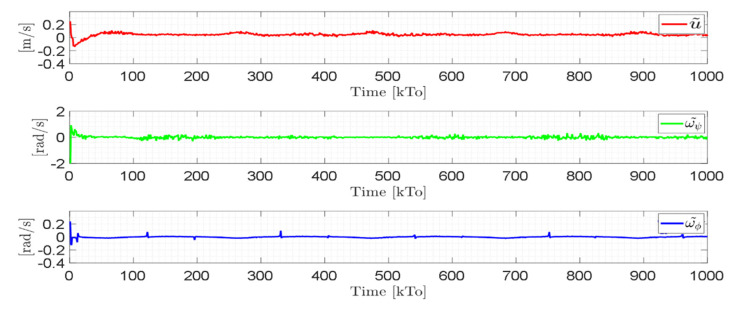
Time evolution of the velocity errors $\tilde{\mu }\left(t\right)=\left(\tilde{u},{\tilde{\omega }}_{\psi },{\tilde{\omega }}_{\phi }\right)$.

The control errors $\tilde{\eta }\left({\tilde{\eta }}_{x},{\tilde{\eta }}_{y},{\tilde{\eta }}_{z}\right)\in R^{3}$ converge to values close to zero asymptotically, i.e., achieving final feature errors $\max\left\|\tilde{\eta }\left(t\right)\right\|<0.06\;[m]$, as shown in Figure 23. Figure 24 shows velocity errors are bound and different from zero $\tilde{\mu }\left(t\right)=\mu_{ref}\left(t\right)-\mu \left(t\right)\neq 0$.

**Figure 25:**
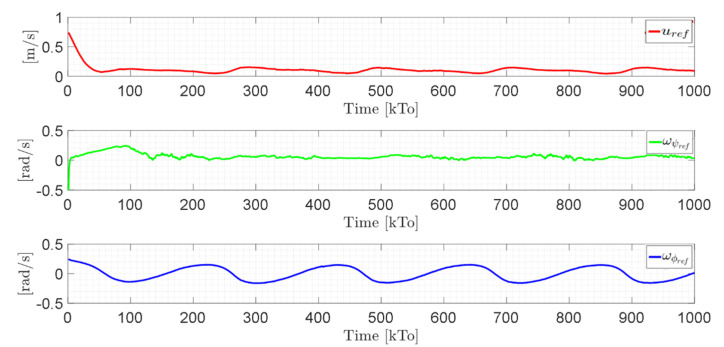
Velocity commands to the standing wheelchair $\mu_{ref}\left(kT_{0}\right)=\left(u_{ref},\omega_{\psi_{ref}},\omega_{\phi_{ref}}\right)$.

Velocity errors are caused by wheel slippage and by frictional forces between the wheelchair and the surface where the tests are being run (virtual environment). Therefore, the velocity errors are limited. In this experiment, the bound of the maximum velocity error is $\max\left\|\tilde{\mu }\left(t\right)\right\|<0.1$. Figure 25 shows the control actions applied to the standing wheelchair robot during the experimental test.

### 7.3. Hardware Performance

The experimental results presented in Section 7 were implemented on the target hardware, according to the mentioned techniques (HIL or FS). For the development of the experiments, a computer with advanced features was used (AMD Ryzen 5 3500×, NVIDIA^®^ GeForce^®^ GTX 1060 video card, 16 GB of RAM, 64-bit Windows 10 operating system), sound sources, and HCT VIVE pro VR glasses.

**Figure 26:**
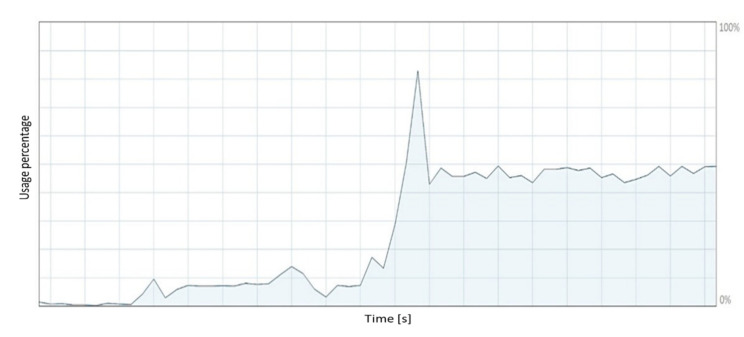
GPU performance.

Figure 26 shows the computational performance of the graphics processing unit (GPU) when running the developed virtual simulator. The computational performance of the GPU briefly reaches 42% of the nominal performance. The moderate consumption of the computational capacity of the graphics card is attributed to the optimization of the graphics resources considered in the external resources design stage detailed in Section 5.

**Figure 27:**
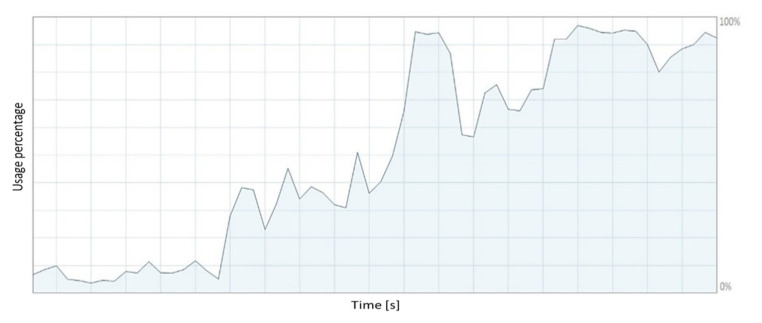
CPU performance.

Figure 27 shows the performance of the central processing unit (CPU) when running the virtual simulator. The CPU performance is around 83% of computational capacity during the simultaneous execution of the Unity and MatLab software when implementing the control algorithms going forward during the experimental tests.

From the results shown in Figure 26 and Figure 27, it can be concluded that the used computer supports the execution of the developed virtual simulator. The computational performance of the computer is below the maximum performance threshold of the components that make up the hardware. The computational performance of the computer has a direct relationship with the execution time of the proposed control schemes.

**Figure 28:**
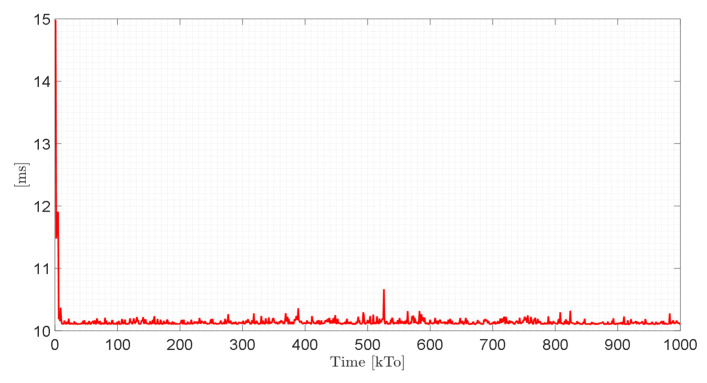
Execution time of the proposed closed-loop control scheme.

It is important to mention that the computational performance of the computer has a direct relationship with the execution time of the proposed control schemes. Therefore, Figure 28 shows the execution time of the control scheme implemented with the FS technique for each sampling time. The machine time in each sampling period is between 10 and 11 (ms). Therefore, it is possible to conclude that the virtual simulator developed allows the execution of autonomous control tasks for a sampling period greater than the machine time.

**Figure 29:**
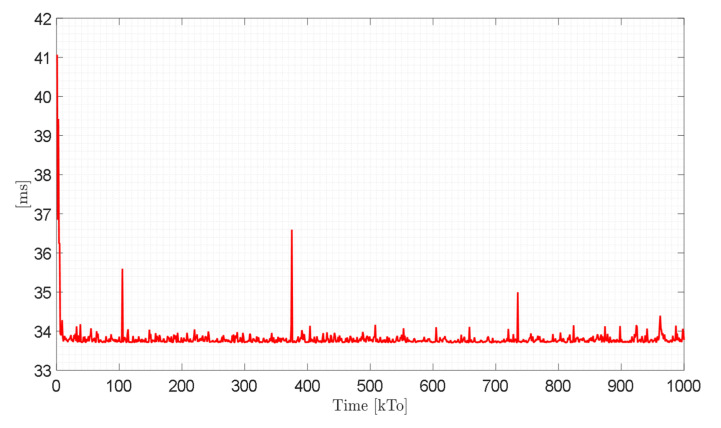
Algorithm execution time during Hardware in the Loop technique.

Finally, Figure 29 shows the execution time of the control scheme implemented with the HIL technique for each sampling time. The peaks observed in the figure correspond to the delay time in the wireless communication between the control unit and the virtual environment developed. It should be noted that for the HIL technique, a Raspberry Pi (Raspberry Pi Foundation, Cambridge, England) as target controller was considered in this work. For the implementation of the HIL technique, it is recommended that the sampling period be $T_{0}\geq 0.5\;[s]$, since the machine time considers the wireless communication between the control unit and the virtual environment. For service robotics applications, specifically in the area of rehabilitation and assistance of person, the velocity of movement of the robot–human system must be low; therefore, considering the Nyquist–Shannon sampling theorem, the sampling time can be greater than $T_{0}\geq 0.5\;[s]$.

### 7.4. Usability of the Simulated System

The usability of the system was analyzed with the help of group of 20 people. The activities of all the participants began with the installation of the developed virtual application. Before the experiments, all participants were trained to navigate VR environments, aimed at leveling the experience in the use of immersive VR environments. In the training, no autonomous control tasks were considered for rehabilitation or robotic assistance. After finishing the experiments, the experimental group completed a usability test to measure the level of acceptance of the system’s features. To measure the degree of usability of the developed application, we used the System Usability Scale (SUS) [40], which is probably the most popular questionnaire to measure the usability attitudes of a system [41]. The total average SUS score obtained was 82.5%, which indicates an excellent degree of usability for our simulator. The designed application, besides being as simple as possible, must also have a high degree of usability.

## 8. Discussion

In this work, a VR-based framework to simulate different control schemes through a robotic wheelchair was developed. The framework also allows the simulation of robotic assistance tasks and the implementation of motor rehabilitation exercises for people with motor disabilities. The latter can be extended to home-based environments, thus favoring tele-rehabilitation. Therefore, people with reduced mobility or motor disabilities can take advantage of this framework, making everyday life easier for them.

Unlike other simulators oriented to the research of robotic systems applied to physical rehabilitation that we can find in the literature, the action of standing and the ability to move while simulating real scenarios has been scarcely explored. In most cases, VR-based physical rehabilitation is performed statically. That is to say, the patient remains in the same place in the rehabilitation session. Although this may be somewhat favorable to maintain the integrity of the person and the robotic system, it has been shown that the sensation of movement can generate rewards that motivate the user to not abandon the training sessions [42]. In addition, one of the most relevant features of the framework deals with the fact that no simulator has explored the range of movements that has direct impact on the patient while performing the movements. This feature is not possible in commercial simulators that do not have the ability to simulate human movements, so this process is essential before, during and after the assistance and rehabilitation. This idea has been considered in the developed simulator, because it allows the avatar to change the position according to the user’s position in real time through cameras or motion capture sensors in the actual implementation of the project.

Within the areas of robotic assistance and rehabilitation, one can define ADL tasks assisted autonomously by a robotic system [43,44]. For this purpose, a robotic standing wheelchair has been considered for the autonomous control of movements of the robot–human system. The work presented in this article falls under the scope of service robots, which work autonomously or semi-autonomously to perform tasks that are useful for the well-being of people [10,45]. In particular, the specific scope is the development of wheelchair prototypes (especially electric wheelchairs) with varying degrees of autonomy designed for people who cannot move their lower and/or upper extremities. Wheelchairs can improve the quality of living for people with motor disabilities, so that people can perform everyday tasks and see the world with other possibilities [11,17].

We find in the literature that different control algorithms have been implemented for the execution of autonomous or semi-autonomous tasks in each prototype developed. The tasks have been developed through vision sensors, audio signals, electromyographic signals (EMGs), electroencephalogram signals (EEGs) and gestural signals, among other signals [10,13,14]. In order to design and implement control algorithms, the physical robotic system is required in order to experimentally evaluate the developed control proposals. To overcome this drawback, different simulation software oriented to robotic systems have been commercially developed [46,47]. However, when considering robotic systems for the rehabilitation area, there is no commercial software or free software that allows one to simulate the behavior of robotic systems oriented to the execution of assistance or rehabilitation tasks. Therefore, the development of new interactive and 3D simulators applied to the area of service robotics is a new trend in the scientific community, whose implementation has been accelerated by the COVID-19 pandemic [4,43,48].

In fact, it is important to mention that the development of this work has been motivated and influenced by the COVID-19 pandemic, which has generated mobility restrictions, making it more difficult for people to attend hospitals, rehabilitation centers, institutes or laboratories to develop experimental tests on robot–human systems. This is why the possibility of being able to simulate rehabilitation and/or robotic assistance tasks in safe conditions prior to their application with patients becomes even more important. The set made up of the standing wheelchair and the framework that we have developed constitutes a very useful rehabilitation technology created in the pandemic. There are other technological developments that have been recently created in different areas of knowledge as a result of the COVID-19 pandemic [49,50]. We firmly believe that these technological developments are likely to last once the pandemic is over [37,51].

The developed VR environment considers both the kinematic and dynamic models of the standing wheelchair. The dynamic model considers the displaced center of mass, which can be caused by poor posture of the person, amputation of the limbs, or spinal injury, etc., which differs from the literature works [4,37]. In addition, the dynamic model considers as input signals the maneuvering velocities of the robotic standing wheelchair, in a similar way as commercial robots do. A trajectory tracking algorithm has been proposed for the autonomous control of the robotic system. The proposed controller design is based on the standing wheelchair kinematic model, in which analytical saturation is implemented in order to limit the maneuverability commands of the robotic system. As far as the trajectory tracking is concerned, we have considered that the desired velocity may depend on the rehabilitation task or robotic assistance. This consideration differs from other works found in the literature, which consider that the desired velocity is constant [9,14,52]. The studies found in the literature have solved the trajectory tracking problem, by choosing the desired velocity equal to the derivative with respect to the time of the desired trajectory [53], which is not logical in autonomous tasks that transport a person with some degree of motor disability. Furthermore, no works were found implementing path-following strategies for standing wheelchairs. We only found works for wheelchairs without the standing degree of freedom [10,52]. Lyapunov’s theory helped us to show that control errors converge to values close to zero, if velocity errors are bound, which confirms that the proposed control scheme works correctly. Therefore, the movements of the standing wheelchair meet the objectives of an autonomous rehabilitation task or robotic assistance.

The experiments carried out in our study showed the performance and versatility of the proposed controller. Furthermore, the obtained usability test results demonstrated a high degree of usability for the developed virtual application [40,54]. Another interesting feature of our system lies in the exchange of information in the bilateral communication between the 3D graphic engine and the mathematical software, whose time is in the microseconds range [37]. Therefore, this fact leads us to consider that our simulator is a real-time simulator, considering that for assistive robotics the sampling period can be greater than 0.05 (s).

The developed framework opens doors to the creation of customized rehabilitation plans with the help of medical experts, as they have the clinical criteria to plan the rehabilitation tasks aimed at every single person with motor disabilities. It is worth mentioning that there is a high risk of muscle injuries when a rehabilitation plan is incorrectly executed, since inadequate movements or abrupt movements may result in muscle injuries, for example [51].

## 9. Conclusions

The framework developed in this work has demonstrated its ability to simulate robotic assistance and motor rehabilitation tasks through a standing wheelchair prior to its implementation with human beings. The simulation techniques used for autonomous control have proven to meet the necessary requirements that these tasks need for a safe operation. Therefore, future work deals with the development and implementation of an autonomous neurorehabilitation plan for people with lower/upper limb motor disabilities. We will take advantages of the benefits of virtual environments for patient rehabilitation as well as the benefits of using sensors. In this sense, we plan to track a person’s movement through the “3 Space Mocap” sensors (YEI Technology, Portsmouth, OH, USA) [55,56]. The conjunction of both technologies will allow the patient to have a good immersive and interactive experience within the virtual environments developed in this work. Our research will also be focused on the development of new control strategies based on the dynamic model of the standing human–wheelchair system for the robotic assistance of people with motor disabilities.

## Figures and Tables

**Table 1:** Implementation of closed-loop control algorithms.

Control System Configuration	Full Simulation (FS)	Rapid Control Prototyping (RCP)	Hardware in the Loop (HIL)	Deployed System (DS)
Control laws and signal processing	Simulated	Simulated	Deployed to target hardware	Deployed to target hardware
Robot, feedback, and power converter	Simulated	Physical components	Simulated	Physical components
Primary benefits	Easy to develop and make changes; full set of analysis tools.	Easy to modify control laws; full set of analysis tools.	Safely and quickly validate deployed control laws	Cost and reliability appropriate for field operation

**Table 2:** Desired task and initial parameters.

Initial Conditions				Desired Task	
$\eta_{0x}$	−10 [m]	$u_{0}$	0 [m/s]	$\eta_{dx}$	2cos(0.05t)−10.58 [m]
$\eta_{0y}$	−11 [m]	$\omega_{\psi 0}$	0 [rad/s]	$\eta_{dy}$	2sin(0.05t)−11.12 [m]
$\eta_{0z}$	0.5 [m]	$\omega_{\phi 0}$	0 [rad/s]	$\eta_{dz}$	0.2sin(0.3t)+0.21 [m]
$\eta_{0\psi }$	0 [rad]	-	-	$v_{\max}$	$0.32\;[\frac{m}{s}]$

## Appendix A

Standing Wheelchair Dynamic Model

$\left[\begin{array}{c} \mu_{ref_{p}}{\left(t\right)}_{2x1}\\ \omega_{\phi_{ref}}{\left(t\right)}_{1x1} \end{array}\right]=\left[\begin{array}{cc} M_{p}{\left(\varsigma \right)}_{2x2} & 0_{2x1}\\ 0_{1x2} & M_{b}{\left(\phi ,\varphi \right)}_{1x1} \end{array}\right]\left[\begin{array}{c} {\dot{\mu }}_{p}\\ {\dot{\omega }}_{\phi } \end{array}\right]+\left[\begin{array}{cc} C_{p}{\left(\varsigma ,\mu_{p}\right)}_{2x2} & 0_{2x1}\\ 0_{1x2} & C_{b}{\left(\phi ,\dot{\phi },\varphi ,\dot{\varphi }\right)}_{1x1} \end{array}\right]\left[\begin{array}{c} \mu_{p}\\ \omega_{\phi } \end{array}\right]+\left[\begin{array}{c} 0_{2x1}\\ g{\left(\phi \right)}_{1x1} \end{array}\right]$

$M_{p}\left(\varsigma \right)=\left[\begin{array}{cc} \varsigma_{1_{p}}+\varsigma_{2_{p}}m_{h} & -\left(\varsigma_{3_{p}}+\varsigma_{4_{p}}m_{h}\right)\\ -\left(\varsigma_{5_{p}}+\varsigma_{6_{p}}m_{h}\right) & \varsigma_{7_{p}}+\varsigma_{8_{p}}m_{h} \end{array}\right];C_{p}\left(\varsigma ,\mu_{p}\right)=\left[\begin{array}{cc} \varsigma_{9_{p}} & \dot{\psi }\left(\varsigma_{10_{p}}+\varsigma_{11_{p}}m_{h}\right)\\ 0 & \varsigma_{12_{p}} \end{array}\right]$

$M_{b}\left(\phi ,\varphi \right)=\left[\varsigma_{13}+\left(\varsigma_{14}+\varsigma_{15}\varphi \right)\frac{\cos{\left(\phi \right)}^{2}}{\sin\left(\phi +\phi_{e}\right)\sin\left(\beta -\phi \right)}\right]$ 

$C_{b}\left(\phi ,\dot{\phi },\varphi ,\dot{\varphi }\right)=\left[\varsigma_{16}+\left(\varsigma_{17}\dot{\varphi }\cos{\left(\phi \right)}^{2}+\left(\varsigma_{20}\omega_{\phi }+\varsigma_{21}\varphi \omega_{\phi }\right)\sin\left(2\phi \right)\right)\frac{1}{\sin{\left(\phi +\phi_{e}\right)}^{2}\sin\left(\beta -\phi \right)}+\left(\varsigma_{18}\omega_{\phi }+\varsigma_{19}\varphi \omega_{\phi }\right)\frac{\cos{\left(\phi \right)}^{2}\cos\left(\phi +\phi_{e}\right)}{\sin\left(\phi +\phi_{e}\right)\sin\left(\beta -\phi \right)}\right]$

$g\left(\phi \right)=\left[\varsigma_{22}g\frac{\cos\left(\phi \right)}{\sin\left(\beta -\phi \right)}\right]$

Dynamic parameters of the standing wheelchair.

$\varsigma_{1}$ = 0.0987; $\varsigma_{2}$ = 0.0046; $\varsigma_{3}$ = 0.0986; $\varsigma_{4}$ = −0.00014; $\varsigma_{5}$ = 0.0987; $\varsigma_{6}$ = −0.0001; $\varsigma_{7}$ = 0.0987; $\varsigma_{8}$ = 0.0032; $\varsigma_{9}$ = 0.9214; $\varsigma_{10}$ = 0.0986; $\varsigma_{11}$ = −0.0019; $\varsigma_{12}$ = 0.9582; $\varsigma_{13}$ = 0.1885; $\varsigma_{14}$ = 0.0214; $\varsigma_{15}$ = −0.0001; $\varsigma_{16}$ = 1.00; $\varsigma_{17}$ = 0.0003; $\varsigma_{18}$ = −0.0085; $\varsigma_{19}$ = −0.0004; $\varsigma_{20}$ = 0.0229; $\varsigma_{21}$ = 0.0005; and $\varsigma_{22}$ = −0.0038.
